# Children’s number line estimation strategies: evidence from bounded and unbounded number line estimation tasks

**DOI:** 10.3389/fpsyg.2024.1421821

**Published:** 2024-11-07

**Authors:** Mengxia Li, Jiahui Yang, Xiaolin Ye

**Affiliations:** School of Teacher Education, Huzhou University, Huzhou, China

**Keywords:** number line estimation, children, strategy utilization, bounded number line estimation, unbounded number line estimation

## Abstract

This study investigates the number line estimation (NLE) strategies utilized by children aged 4–7 across both bounded and unbounded NLE tasks. Drawing on prior research, it hypothesized that younger children would predominantly employ benchmark-based strategies, with endpoints as reference points, in bounded tasks, while older children would utilize a wider range of reference points including midpoints and quartiles. For unbounded tasks, it was anticipated that both younger and older children would adopt the scalloped strategy. A total of 181 Chinese children participated, representing three educational backgrounds: Middle Class (kindergarten), Senior Class (kindergarten), and Grade 1 (elementary school). They completed the bounded and unbounded NLE tasks with numbers ranging from 0 to 50. Data analysis focused on estimation accuracy using Percent Absolute Error (PAE) and contour analyses to examine strategy use. Results revealed that all age groups employed benchmark-based strategies using endpoints and midpoints in both bounded and unbounded tasks, and applied the scalloped strategy with units of integers 5 or 10 across all tasks. Findings suggest a coexistence of benchmark-based and scalloped strategies across task types, reflecting children’s intuitive estimation strategies. Furthermore, children aged 4 to 7 exhibited consistent strategy utilization, indicating a developmental stage characterized by reliance on specific reference points for estimation. This study contributes to understanding the developmental trajectory of number line estimation strategies in early childhood and emphasizes the importance of task type, learning experiences, and other factors in eliciting different estimation strategies.

## 1 Introduction

Number line estimation (NLE) is essential for understanding numerical cognition and provides valuable insights into broader cognitive development. It reveals how children perceive and interpret numerical magnitudes, which form the basis for more advanced mathematical reasoning and problem-solving skills (Siegler and Booth, 2004). NLE is often used as a measure of an individual’s ability to represent numbers spatially. Consequently, performance on NLE tasks is commonly utilized as an indicator of developmental progress in number representation throughout childhood. During these tasks, children employ diverse estimation strategies to approximate the position of target numbers on a number line or approximate the number of target position on a number line. Siegler and Booth (2004) proposed that age-related increases and accumulated experience account for enhancements in the precision of NLE and the advancement of representational models. Moreover, the strategies employed by children during NLE tasks offer insights into how age and experience contribute to the refinement of numerical estimation skills.

In NLE tasks, participants are tasked with estimating the spatial position of specific numbers within a predefined numerical range, such as 0–10 or 0–100. This task has been extensively explored in previous research (e.g., Siegler and Opfer, 2003; Berteletti et al., 2010; Booth and Siegler, 2006, 2008; Ebersbach et al., 2015; Siegler and Booth, 2004; Siegler and Opfer, 2003; Thompson and Opfer, 2010). More recently, researchers have increasingly focused on unbounded NLE tasks, which are posited to provide a more accurate reflection of the developmental state of numerical representations (Cohen and Blanc-Goldhammer, 2011; Link et al., 2014; Peeters et al., 2015; Reinert et al., 2019; Jung et al., 2020). In unbounded NLE, the number line is presented without defined end points, except for a marked unit length (e.g., 0–1) and the origin. Participants must then place target numbers along this continuum, starting from the defined unit length. This shift from bounded task to both bounded and unbounded NLE tasks marks a critical development in assessing numerical cognition.

In NLE tasks, children often employ different estimation strategies that evolve with age. Research indicates that individuals in early childhood may use counting-like strategies, also referred to as cumulative strategies, within these tasks. With increasing age, both the frequency and diversity of strategy usage increase (Schneider et al., 2018). Particularly, preschool children and those in the early grades of elementary school, who possess limited mathematical learning experience, are more likely to rely on counting-like strategies. As numerical cognition develops, these strategies in bounded NLE tasks transition to more sophisticated estimation methods, such as benchmark-based strategies (Ashcraft and Moore, 2012; Barth and Paladino, 2011; Peeters et al., 2015; Rouder and Geary, 2014). Benchmark-based strategies also known as the proportion judgment strategy or anchor-based strategy, involve the use of reference points, including the origin, end, midpoint, and quartiles of the number line.

Researchers have identified three progressive stages in the adoption of benchmark-based strategies among children: initially, children use solely the origin as a reference point. In the subsequent stage, they incorporate all available markers on the line, such as both the origin and ending points. In the final stage, children extend their reference to include the midpoint and quartiles, enhancing their estimation accuracy (Luwel et al., 2019). Conversely, in unbounded NLE tasks, the adoption of the scalloped strategy becomes more prevalent. This strategy, which relies on integer estimation, prompts children to select certain “estimation units,” such as increments of 5 or 10, to facilitate their estimations. This method fosters a “scalloped power model,” sometimes termed the “dead-reckoning strategy,” which is characterized by segmental and incremental estimation (Cohen and Blanc-Goldhammer, 2011; Cohen and Sarnecka, 2014; Link et al., 2014; Jung et al., 2020).

The strategies employed by children in NLE tasks are influenced by both the numerical range presented and their familiarity with numbers. As children age, they begin to selectively adopt estimation strategies that are appropriate to the experimental requirements (Schneider et al., 2018). For instance, in bounded NLE tasks within a 0 to 100 range, 5-year-old children typically use reference points such as the origin and endpoint. By the age of 7, children not only utilize these reference points but also further segment the line using the midpoint, indicating an enhancement in their strategic approach (Barth and Paladino, 2011). Similarly, studies indicate that within a smaller range, such as 0–20, 6-year-olds primarily rely on the origin point as a reference. By the age of 7, these children not only recognize the endpoints as reference points but also begin to internally generate a midpoint. This progression in strategy usage becomes even more pronounced by the age of 8, as children’s ability to internally structure the number line matures (White and Szűcs, 2012).

Research contrasting bounded and unbounded NLE tasks has shown distinct strategy utilizations among children aged 3.6–8 years. In bounded NLE tasks, children employ benchmark-based strategies, using reference points such as endpoints and midpoints, whereas in unbounded NLE tasks within the number ranges of 0–20 and 0–100, they adopt the scalloped strategy (Cohen and Sarnecka, 2014; Ebersbach et al., 2015). Notably, the use of strategies in bounded NLE tasks tends to increase with age, unlike in unbounded NLE tasks, where strategy adoption did not exhibit significant age-related variation (Cohen and Sarnecka, 2014). Further investigations have indicated that children of age 8 and above apply different estimation units across various tasks, leading to “single, double, and multiple scalloped strategies” (Jung et al., 2020). Link et al. (2014) compared the developmental differences in bounded and unbounded NLE tasks among children in grades 1 through 4 (ages 7–10) and adults. The results revealed that children in grades1 and 2 exhibited similar performance in both bounded and unbounded NLE tasks, with no evidence of benchmark-based or scalloped strategies. It wasn’t until grade 3 that benchmark-based strategies emerged in the bounded NLE task. Additionally, a study examining NLE strategies within the 0–100 range among children from middle kindergarten to second grade revealed that none of the groups consistently used reference point strategies in either bounded or unbounded NLE tasks. This underscores the significant role that number familiarity plays in the effectiveness of NLE performance (Ebersbach et al., 2015).

Existing research has primarily concentrated on bounded and unbounded NLE strategies among children aged 7 and above, as well as adults (Reinert and Moeller, 2021). However, investigations into estimation strategies for younger age groups in both bounded and unbounded tasks are scant and yield inconsistent results, rendering the strategies employed by children under 7 in both types of NLE tasks ambiguous. Younger children may not effectively use the endpoints of the number line as reference points due to their limited numerical knowledge, in contrast to older children who might already grasp the significance of these endpoints. Consequently, younger children may adopt different estimation strategies compared to their older counterparts. Notably, by age 4, children begin to understand the relationship between counting and number positioning, demonstrating an interest in numerical sequences and quantitative relationships. This underscores the necessity for further research into the strategies employed by children aged 4 to 7 in NLE tasks.

The present study aims to investigate the strategies utilized by children from middle kindergarten (age 4) to grade 1 (age 7) in elementary school across both bounded and unbounded NLE tasks. Building on prior research, we hypothesized that in bounded NLE tasks, younger children would utilize benchmark-based strategies with endpoints serving as reference points, whereas older children would employ a wider range of reference points, including endpoints, midpoints, and quartiles. In unbounded NLE tasks, it was anticipated that both younger and older children would adopt the scalloped strategy.

## 2 Methods

### 2.1 Participants

A total of 181 Chinese children were enrolled in the study from three different educational backgrounds in Mainland China: Middle Class (61 children: 29 girls and 32 boys, aged 56.69 ± 3.53 months), Senior Class (60 children: 27 girls and 33 boys, aged 66.18 ± 3.84 months) in kindergarten, and Grade 1 (60 children: 31 girls and 29 boys, aged 67.24 ± 3.36 months) in elementary school. All participants had acquired the ability to recognize numbers up to 50, either through instruction at home or in their respective kindergartens. The study was conducted with approval from the Academic Ethics Committee of Huzhou University. Parental consent was secured before the commencement of testing, and participants were incentivized with a gift valued at 20 RMB.

### 2.2 Experimental tasks and materials

Drawing from the experimental design of Kim and Opfer (2017, 2020), where participant ages ranged from 4.25 to 12.21 years, and number line ranges for bounded and unbounded NLE tasks varied extensively, our study tailored the numerical materials accordingly. A pilot study was conducted involving eight randomly selected children from the middle class to refine the experimental parameters. Based on the outcomes of this preliminary assessment, a number line ranging from 0 to 50 was selected for both bounded and unbounded NLE tasks in the study. This smaller range better aligns with the numerical familiarity of younger children aged 4–7, as they tend to have a more robust understanding of numbers in this range. Previous studies (e.g., Ebersbach et al., 2015) suggest that children perform better with familiar ranges, making the 0–50 range suitable for more accurate assessments, reducing confusion, and enhancing engagement during the tasks.

For the bounded NLE task, specific Arabic numerals were utilized: 1, 3, 6, 10, 13, 15, 19, 25, 31, 35, 38, 40, 44, and 49. During each trial, participants were clearly informed that the target number would shift horizontally along a number line, which was clearly marked with an origin point at 0 and an endpoint at 50. Participants were tasked with estimating the position of each given number on this scale. To ensure robust data, each number was presented twice to the participants.

In the unbounded NLE task, participants were presented with the same 0–50 number line and the same frequency of numerical material repetitions as in the bounded condition. However, unlike in the bounded task, the endpoint of the number line was not delineated. Participants were required to assess the position of a given number using the unit length, defined as the distance from 0 to 1. The estimation process involved the same Arabic numerals materials as those used in the bounded NLE, with the exception of the number “1.” The number “3” was designated as the origin point for the unbounded number line.

### 2.3 Experimental procedure

The NLE tasks were presented on an 11-inch PAD, with the screen measuring 247.60 mm in width. For both bounded and unbounded NLE tasks, the number lines extended to a total length of 198.08 mm. The experimental design included both practice and formal experiments, with feedback provided during the practice sessions. In the formal experiments, participants were instructed to estimate the position of the given number on the number line by touching the screen. After marking their estimation, they were to click “confirm” and “continue” to advance to the subsequent trial.

In the unbounded NLE tasks, the absence of an endpoint on the line was compensated by the inclusion of a unit mark “1” in the lower-left corner of the screen. Apart from this modification, all other aspects were consistent with those in the bounded NLE tasks, as described in Link et al. (2014, Figure 1). To avoid the influence of the endpoints used in the bounded NLE tasks on participants’ performance in the unbounded NLE tasks, children first complete the unbounded NLE task. A two-day interval was then enforced before proceeding to the bounded NLE task.

**Figure 1:**
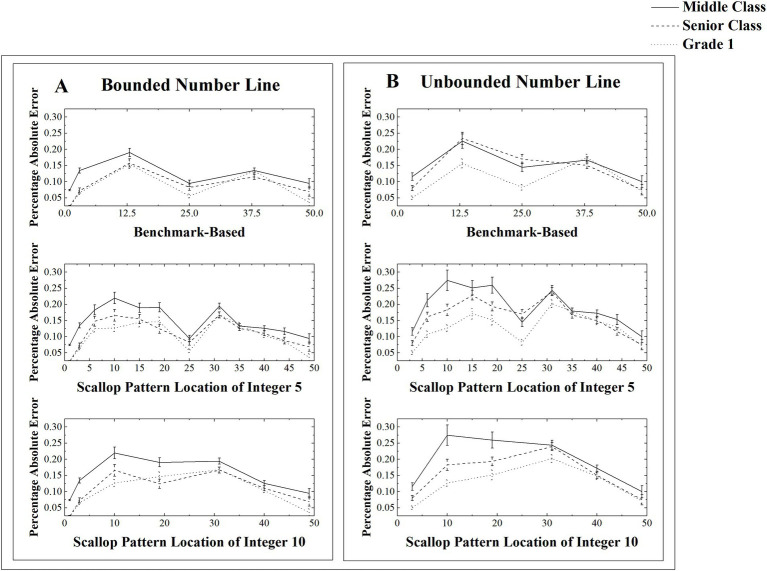
Mean Percentage of absolute error (and standard errors) at the strategy point as a function of group and tasks (A for bounded NLE tasks, B for unbounded NLE tasks). The top panel represents reference points based on the benchmark-based strategy, the middle panel represents the units’ points based on the scallop strategy with units of 5, and the bottom panel represents the units’ points based on the scallop strategy with units of 10. ‘Middle Class’ refers to kindergarten children aged 4–5 years, ‘Senior Class’ refers to kindergarten children aged 5–6 years, and ‘Grade 1’ refers to elementary school children aged 6–7 years.

### 2.4 Data analysis

#### 2.4.1 Estimation accuracy and strategy use

In previous research, the Percent Absolute Error (PAE) has been employed as a dependent variable to analyze children’s strategies in NLE tasks (Booth and Siegler, 2008; Jung et al., 2020; Link et al., 2014; Slusser and Barth, 2017). PAE is calculated using the formula: PAE = |estimated value – actual value|/number line range, which facilitates the comparison of estimation accuracy across different conditions and groups. As a result, the smaller the PAE value, the higher the estimation accuracy.

To assess the utilization of benchmark-based strategies, contour analyses were performed for each child group. These analyses aimed to contrast the distribution of estimation errors around the reference points (origin, end, mid, and quartile points) on the number line. This approach provides insights into how children use these benchmarks to guide their estimations in both bounded and unbounded NLE tasks (Ashcraft and Moore, 2012; Jung et al., 2020). Specifically, estimates for numbers such as those starting from the endpoints (both origin point and endpoint; with the origin point designated as “1” in bounded tasks and “3” in unbounded tasks), the midpoint (25), and quartiles (23, 38) were aggregated to reflect the use of benchmark-based reference points (cf. Jung et al., 2020; Lai et al., 2018).

Similarly, to examine the adoption of scalloped strategies, contour analyses were also conducted for each child group focusing on estimation errors around key integer reference points (5 and 10). This method served as an index of children’s use of scalloped strategies in both bounded and unbounded NLE tasks (Ashcraft and Moore, 2012; Jung et al., 2020). Estimates for numbers typically associated with scalloped reference points (6, 10, 15, 19, 25, 31, 35, 40, 44, and 49) were pooled to show the strategic use of these points (cf. Cohen and Blanc-Goldhammer, 2011).

## 3 Results

### 3.1 Number line estimation accuracy

Table 1 presents the mean PAE of the three groups of children. A 3 (group: Middle Class vs. Senior Class vs. Grade 1) × 2 (Tasks: bounded vs. unbounded NLE task) ANOVA was conducted on the mean PAE. The results revealed a significant main effect for group [*F*(2, 177) = 3.98, *p* = 0.027, $\eta_{p}^{2}$ = 0.15], with PAE of children from middle class was higher than those of grade 1 (*t* = 2.80, *p* = 0.024). There was also a significant main effect for task [*F*(1, 177) = 72.36, *p* < 0.001, $\eta_{p}^{2}$ = 0.11], with PAE in unbounded NLE task was higher than that of bounded NLE (*t* = 2.80, *p* = 0.024). Additionally, a marginally significant interaction between task and group [*F*(3, 177) = 2.99, *p* = 0.063, $\eta_{p}^{2}$ = 0.009]. Further simple effect analysis revealed that the mean PAEs of children from middle classes was significantly larger than those of grade 1 in unbounded NLE task (*t* = 3.18, *p* = 0.030). These findings suggest that older children’s strategy use in NLE is more effective than that of younger children. Specifically, strategy use in bounded NLE tasks is superior to that in unbounded tasks, and within the unbounded tasks, the strategy use of children in Grade 1 surpasses that of children in the Middle Class. No significant difference was found between the Senior Class and the other two groups.

**Table 1:** Mean PAE’s and SD of PAE’s of bounded and unbounded NLE tasks for difference age groups.

Task	Group	*N*	Mean	SD
Bounded NLE	1	61	0.154	0.042
	2	60	0.122	0.036
	3	60	0.114	0.041
Unbounded NLE	1	61	0.192	0.057
	2	60	0.165	0.053
	3	60	0.135	0.046

### 3.2 Number line estimation strategies use

#### 3.2.1 Benchmark-based strategies use

Table 2 presents the mean PAE in different reference points of the three groups of children. The contour analyses were conducted to assess the benchmark-based strategies using the PAE around the benchmark-based reference points (Ashcraft and Moore, 2012). We averaged the PAE of the origin and ending point as endpoints. A 3 (group: Middle Class vs. Senior Class vs. Grade 1) × 2 (Tasks: bounded vs. unbounded NLE task) × 4 (reference point: endpoints vs. midpoint, vs. quartile vs. others) ANOVA on the mean PAE was conducted (see Figure 1 top panel). The results revealed a significant main effect for group [*F*(2, 177) = 14.32, *p* < 0.001, $\eta_{p}^{2}$ = 0.32], with PAE of children from middle class was higher than those of senior class (*t* = 2.27, *p* = 0.025), and senior was higher than those of grade 1 (*t* = 3.07, *p* = 0.005). A significant main effect for task [*F*(1, 177) = 48.37, *p* < 0.001, $\eta_{p}^{2}$ = 0.04], with PAE in unbounded NLE task was higher than that of bounded NLE (*t* = 6.96, *p* < 0.001). A significant main effect for reference point [*F*(4, 177) = 128.48, *p* < 0.001, $\eta_{p}^{2}$ = 0.18], with PAE of endpoints was lower than that of midpoint (*t* = 6.96, *p* < 0.001) and quartile (*t* = 17.25, *p* < 0.001), and non-reference points (*t* = 15.46, *p* < 0.001). PAE of midpoint was lower than that of quartile (*t* = 10.49, *p* < 0.001) and non-reference point (*t* = 8.69, *p* < 0.001). The main effect for the reference point indicates the use of a benchmark-based strategy, with the effect of reference points diminishing from endpoints to midpoints and then to quartiles.

**Table 2:** Mean PAE’s and SD of PAE’s of reference points in bounded and unbounded NLE tasks for difference age groups.

Reference	Group	Bounded NLE task		Unbounded NLE task	
		Mean	SD	Mean	SD
Endpoints	1	0.084	0.015	0.091	0.017
	2	0.046	0.008	0.075	0.012
	3	0.029	0.006	0.07	0.009
Midpoint	1	0.095	0.012	0.144	0.014
	2	0.083	0.01	0.17	0.015
	3	0.056	0.006	0.082	0.009
Quartiles	1	0.163	0.011	0.194	0.011
	2	0.136	0.007	0.192	0.01
	3	0.143	0.008	0.164	0.014
Others	1	0.165	0.008	0.207	0.014
	2	0.128	0.011	0.169	0.007
	3	0.122	0.007	0.14	0.007

The results revealed a significant interaction between reference point and group [*F*(6, 531) = 3.36, *p* = 0.002, $\eta_{p}^{2}$ = 0.009]. Further simple effect analysis revealed that, among the three group, besides the lack of difference in PAE between quartiles and non-reference points (*Ps* > 0.05), differences exist among the remaining reference points as well as between reference and non-reference points. The results also revealed a significant interaction between reference point and task [*F*(3, 36) = 3.49, *p* = 0.016, $\eta_{p}^{2}$ = 0.003]. Further simple effect analysis revealed that, besides the lack of difference in PAE between quartiles and non-reference points (*Ps* > 0.05), differences exist among the remaining reference points as well as between reference and non-reference points in both bounded and unbounded tasks (*Ps* < 0.005). This implied that none of the three groups of children utilized the quartile strategy.

#### 3.2.2 Scalloped strategies use

We averaged the PAE of estimation number around multiples of 5 or 5 itself as integer 5, and the PAE of estimation number around multiples of 10 or 10 itself as integer 10. Table 3 presents the mean PAE in different unite of the three groups of children. The contour analyses of scalloped strategies were conducted with a 3 (group: Middle Class vs. Senior Class vs. Grade 1) × 2 (Tasks: bounded vs. unbounded NLE task) × 3 (unite: integer 5 vs. integer 10 vs. others) ANOVA on the mean PAE (see Figure 1 middle and bottom panels). The results revealed a significant main effect for group [*F*(2, 177) = 14.56, *p* < 0.001, $\eta_{p}^{2}$ = 0.07], with PAE of children from middle class was higher than those of senior class (*t* = 3.48, *p* = 0.001) and grade 1 (*t* = 5.31, *p* < 0.001). The results revealed a significant main effect for task [*F*(1, 177) = 63.35, *p* < 0.001, $\eta_{p}^{2}$ = 0.07], with PAE in unbounded NLE task was higher than that of bounded NLE (*t* = 7.95, *p* < 0.001). The results also revealed a significant main effect for unite [*F*(1, 177) = 28.35, *p* < 0.001, $\eta_{p}^{2}$ = 0.01], with PAE in non-reference points were higher than that of integer 5 (*t* = 7.23, *p* < 0.001) and 10 (*t* = 9.01, *p* < 0.001).

**Table 3:** Mean PAE’s and SD of PAE’s of unite in bounded and unbounded NLE tasks for difference age groups.

Unite	Group	Bounded NLE task		Unbounded NLE task	
		Mean	SD	Mean	SD
Integer 5	1	0.152	0.01	0.199	0.012
	2	0.124	0.007	0.167	0.007
	3	0.113	0.005	0.138	0.006
Integer 10	1	0.162	0.012	0.21	0.013
	2	0.127	0.008	0.1168	0.007
	3	0.116	0.006	0.141	0.007
Others	1	0.133	0.011	0.168	0.011
	2	0.092	0.005	0.154	0.009
	3	0.095	0.004	0.127	0.008

The interaction of reference point and task, unit and task, and the three-way interaction among unite and group and task were not significant (*ps* > 0.05). These results indicated that all three group of children applied the same scalloped strategies with the unit of integer 5 and integer 10 in both bounded and unbounded NLE tasks.

## 4 Discussion

To investigate the strategies used by children aged 4 to 7 in NLE, we conducted both bounded and unbounded NLE tasks. We measured children’s percent absolute error (PAE) and conducted contour analysis. Previous research, such as Slusser et al. (2013), found evidence of 7-year-olds using benchmark-based strategies in bounded tasks, while Cohen and Sarnecka (2014) found evidence of 3.8-year-olds using the scalloped strategy in unbounded tasks (cf. Reinert and Moeller, 2021). We compared estimation strategy differences between these task versions in a cross-sectional design involving children from middle class of kindergarten to grade 1 of elementary school.

We hypothesized that in bounded NLE tasks, younger children would employ benchmark-based strategies using endpoints as reference points, while older children would use a broader array of reference points, including endpoints, midpoint and quartiles. In unbounded NLE tasks, both younger and older children were expected to use the scalloped strategy. The current results partially confirmed our hypothesis that children used the benchmark-based strategies and scalloped strategies in both bounded and unbounded NLE tasks. Specifically, in both types of tasks, all three groups of children employed benchmark-based strategies using endpoints and midpoints as reference points, but none used quartiles. These results also indicated that all three groups of children applied the same scalloped strategies with unit of integer 5 or 10 in both bounded and unbounded NLE tasks.

Contrary to our hypothesis, we found the benchmark-based strategies in the unbounded NLE task. Specifically, children from middle class and senior class in kindergarten, as well as grade 1 in elementary school used the benchmark-based strategies with the reference of endpoints and midpoint. In the bounded NLE task, the scalloped strategy was observed. Specifically, all three groups of children applied the same scalloped strategy with using unit of integer 5 or 10.

### 4.1 Coexistence of benchmark-based and scalloped strategies in both bounded and unbounded NLE tasks of early childhood

Children may employ multiple strategies simultaneously for NLE, influenced by task demands, cognitive development levels, and learning experiences. Two main types of strategies are observed: intuitive estimation strategies (Schneider and Siegler, 2010) and exact calculation reliance strategies (Mix et al., 1997). Intuitive strategies, such as benchmark or anchor points (Barth and Paladino, 2011), skip counting (Siegler and Booth, 2004), decomposition (Opfer and Siegler, 2007), and endpoint estimation, leverage children’s intuition and everyday experiences to estimate numbers quickly without precise calculations. In contrast, exact calculation reliance strategies involve rigorous mathematical operations and precise calculations, including direct calculation (Gilmore and Papadatou-Pastou, 2009), approximate calculation (Siegler and Opfer, 2003), cumulative summation (Ashcraft and Moore, 2012), and proportional reasoning (Boyer et al., 2008), to determine the position or value on a number line.

In NLE tasks, as individuals grow older, they tend to rely more on precise calculation strategies and decrease the use of intuitive strategies (Siegler, 1987). This trend may reflect the development of cognitive abilities and mathematical skills, as individuals become more capable of utilizing more sophisticated calculation techniques and mathematical concepts for numerical estimation as they age (Booth and Siegler, 2006). However, some studies have found that increasing age does not necessarily lead to a decrease in intuitive estimation strategies but is accompanied by greater strategy diversity and flexibility (Opfer and Siegler, 2007).

Children demonstrate flexibility in using multiple estimation strategies when completing NLE tasks. For instance, they may employ both intuitive estimation strategies based on reference points and precise calculations to validate their estimates within the same task. Additionally, they may combine different types of intuitive estimation strategies, such as simultaneous use of skip counting and decomposition, to achieve a comprehensive estimation result. Children adjust their strategies based on problem complexity, favoring intuitive estimation for simpler problems, and relying more on precise calculations for complex ones. In bounded NLE tasks, children may utilize various estimation strategies, including intuitive estimation based on reference points, decomposition, skip counting, and direct calculation (Opfer and Siegler, 2007). Similarly, in unbounded NLE tasks, children may employ skip counting and proportional reasoning (Siegler and Booth, 2004).

Partially aligning with prior studies, our investigation revealed that children employed benchmark-based and scalloped strategies in both bounded and unbounded NLE tasks. Previous literature has reported the use of benchmark-based strategies, focusing on endpoints and midpoints in bounded tasks, and the scalloped strategy in unbounded tasks (Bailey et al., 2012; Barth and Paladino, 2011; Cohen and Sarnecka, 2014; Ebersbach et al., 2015; Schneider et al., 2018; White and Szűcs, 2012). Our findings suggest that children consistently apply these strategies across different task types. This is because, although benchmark-based and scalloped strategies are often discussed separately in existing research, they both belong to the category of intuitive estimation strategy, relying on intuition and perception rather than precise calculation, thus they share an inherent connection. Firstly, both strategies rely on reference points for estimation. Benchmark-based strategies use specific reference points such as starting points, endpoints, or midpoints, while scalloped strategies divide the number line into several sections, with each section serving as a reference point. Secondly, both strategies partition the number line into local regions and utilize these regions for estimation. Benchmark-based strategies divide the number line into segments and select a reference point within each segment for estimation, while scalloped strategies divide the number line into multiple equal parts and estimate within each part. Therefore, although benchmark-based and scalloped strategies emerged in all three groups of children in both bounded and unbounded NLE tasks, they actually reflect the utilization of intuitive estimation strategies.

### 4.2 Stages of NLE strategy development from middle class to grade 1

Previous research has delineated the progression of children’s utilization of benchmark-based strategies in bounded NLE tasks into three distinct stages. In the first stage, children solely rely primarily on the origin point as a reference. In the second stage, they incorporate all available points on the line, including the origin and endpoint. By the third stage, children not only utilize the provided points but also consider additional landmarks such as the midpoint and quartiles (Luwel et al., 2019). However, in unbounded NLE tasks, the use of the scalloped strategy does not exhibit age-related increases. Our study identified children aged 4–7 as being in the second stage of benchmark-based strategy utilization, wherein they are capable of referencing both endpoints and midpoints for numerical line estimation. Furthermore, their application of the scalloped strategy was characterized by units of integers 5 or 10. In our study, children in the second stage are younger compared to those in Western studies because Chinese children’s numerical development in the early stages surpasses that of their Western counterparts (Stigler and Perry, 1988). Chinese children from middle class of kindergarten not only recognize numbers from 0 to 50 but can even perform addition and subtraction within 10 or 20.

Consistent with Cohen and Sarnecka (2014), children’s strategy utilization in unbounded NLE tasks does not vary with age. Consistent with the findings of Link et al. (2014), our study also confirmed consistent performance of children aged 4–7 years in both bounded and unbounded number line tasks. This consistency may be attributed to their inability, between ages 4 and 7, to distinguish between a line with both an origin and an endpoint and one with only an origin. Hence, for children in this age group, bounded and unbounded NLE tasks appear to carry the same significance (Link et al., 2014). Additionally, this similarity may be influenced by their familiarity with the 0–50 numerical range, which may have allowed them to apply strategies typically reserved for bounded tasks, even in unbounded contexts. For example, instead of using the hypothesized scalloped strategy, children also demonstrated evidence of employing benchmark-based strategies, such as referencing midpoints and endpoints. The choice of a smaller number range, such as 0–50, might have made it easier for children to intuitively identify and use reference points, thus blurring the distinction between bounded and unbounded estimation strategies. Typically, formal education begins around the age of 7 in China, coinciding with entry into primary school. Subsequently, children gradually develop a deeper understanding of abstract mathematical concepts, such as line segments and rays, between ages 7 and 9. This developmental milestone often corresponds with the introduction of more complex mathematical concepts, including geometric properties and classifications, leading to a more systematic exploration of these concepts in their mathematics curriculum.

Compared to previous research, our results suggest that bounded NLE tasks appear to elicit the use of benchmark-based and scalloped strategies in children. These data from children under 7 years old complement recent research evidence, which has mostly focused on studying strategies in bounded NLE tasks in children over 7 years old or the differences between strategies in bounded and unbounded NLE tasks (Cohen and Blanc-Goldhammer, 2011; Cohen and Sarnecka, 2014; Jung et al., 2020; Kim and Opfer, 2017, 2020; Link et al., 2014; Jung et al., 2020). Older children showed a separation of strategies between the bounded and unbounded versions of the task, but we did not find systematic differences between bounded and unbounded NLE in children of 4–7 study. Therefore, for relatively younger children, both versions of the task may have similar performance in NLE, while for older children, the performance in the bounded version may be supplemented by strategies other than NLE. This is particularly interesting from a developmental perspective, suggesting an age-related turning point from consistency to divergence between the two versions of the task in children’s development.

### 4.3 Cross-cultural insights and methodological challenges of unbounded NLE

This study contributes to the understanding of NLE strategies among children aged 4–7 by confirming the use of similar strategies across both bounded and unbounded tasks. This finding highlights the developmental stage where children rely on reference points like midpoints and endpoints, reflecting a consistent approach to NLE regardless of task version. Additionally, our study revisits an important discussion about the early mathematical abilities of Chinese children compared to their Western counterparts. Consistent with previous research (Stigler and Perry, 1988), our findings suggest that Chinese children demonstrate a more advanced understanding of numerical concepts at younger ages, which may influence their performance in estimation tasks. This renewed perspective on cultural differences in mathematical development underscores the need for further comparative studies, offering valuable insights into how cultural and educational contexts shape early numerical cognition.

Although we observed the coexistence of both benchmark-based and scalloped strategies, it remains unclear whether children switch strategies on a trial-by-trial basis or prefer certain ones for specific numerical values (e.g., higher numbers). Siegler’s overlapping waves theory suggests that children may use multiple strategies simultaneously and refine their strategy with experience (Siegler, 2000). Similarly, Siegler’s theory of number development indicates that children’s numerical understanding and strategy use evolve as they face different challenges (Siegler, 2000). This underscores the need for further research into how children adjust their strategies during estimation tasks. Eye-tracking could help determine whether children focus on specific reference points or numerical intervals, providing deeper insights into the real-time application of strategies like the scalloped or benchmark-based approaches (Reinert et al., 2015).

Previous studies have often used one-and two-cycle power functions to model children’s estimation patterns (Barth and Paladino, 2011; Cohen and Blanc-Goldhammer, 2011), demonstrating that these models provide a better fit than logarithmic and linear functions (Reinert et al., 2015; Rouder and Geary, 2014; Slusser et al., 2013). In contrast, our study focused on examining the practical estimation strategies children employ. Future research could explore the relationship between these intuitive strategies and power function models for a more integrated understanding.

Research on unbounded NLE remains in its early stages, with challenges in interpreting results. For example, children might use the edge of the PAD screen as a reference point during unbounded tasks, potentially skewing their estimations. Additionally, children aged 4–7 may struggle to differentiate between bounded segments and unbounded rays, leading to similar performance across both task types. To improve result reliability in unbounded NLE tasks, it is essential to leave adequate space beyond the number line to account for overestimations and adjust screen positioning to reduce reliance on external landmarks (Cohen and Ray, 2020).

## Data Availability

The raw data supporting the conclusions of this article will be made available by the authors, without undue reservation.
